# Epigenetic Regulations of Microglia/Macrophage Polarization in Ischemic Stroke

**DOI:** 10.3389/fnmol.2021.697416

**Published:** 2021-10-11

**Authors:** Meiqian Qiu, En Xu, Lixuan Zhan

**Affiliations:** Institute of Neurosciences and Department of Neurology of the Second Affiliated Hospital of Guangzhou Medical University and Key Laboratory of Neurogenetics and Channelopathies of Guangdong Province and the Ministry of Education of China, Guangzhou, China

**Keywords:** ischemic stroke, epigenetics, neuroinflammation, microRNAs, histone modifications, microglia/macrophage polarization

## Abstract

Ischemic stroke is one of the leading causes of death and disability worldwide. Microglia/macrophages (MMs)-mediated neuroinflammation contributes significantly to the pathological process of ischemic brain injury. Microglia, serving as resident innate immune cells in the central nervous system, undergo pro-inflammatory phenotype or anti-inflammatory phenotype in response to the microenvironmental changes after cerebral ischemia. Emerging evidence suggests that epigenetics modifications, reversible modifications of the phenotype without changing the DNA sequence, could play a pivotal role in regulation of MM polarization. However, the knowledge of the mechanism of epigenetic regulations of MM polarization after cerebral ischemia is still limited. In this review, we present the recent advances in the mechanisms of epigenetics involved in regulating MM polarization, including histone modification, non-coding RNA, and DNA methylation. In addition, we discuss the potential of epigenetic-mediated MM polarization as diagnostic and therapeutic targets for ischemic stroke. It is valuable to identify the underlying mechanisms between epigenetics and MM polarization, which may provide a promising treatment strategy for neuronal damage after cerebral ischemia.

## Introduction

Ischemic stroke, one of the leading life-threatening cerebral disorders (Benjamin et al., 2018), occurs as a result of the reduction or interruption of the blood flow in arteries supplying the brain. To date, the treatment of acute ischemic stroke still largely depends on the intravenous thrombolysis and endovascular treatment (Goyal et al., 2016; Eskey et al., 2018; Thiebaut et al., 2018). Unfortunately, many neuroprotective drugs have failed to show beneficial effects in the treatment of acute ischemic stroke (Auriel and Bornstein, 2010; Xu and Pan, 2013; Kikuchi et al., 2014; Dhir et al., 2020). Consequently, there is a pressing need to conduct further research to clarify the mechanism of ischemic brain injury and to find effective targets for the diagnosis and treatment of ischemic stroke.

Microglia/macrophages (MMs) are the first line of defense against central nervous system (CNS) injuries, and they function as a key participant in maintaining brain homeostasis. The resident microglia and peripheral macrophages are rapidly activated after cerebral ischemia (Perego et al., 2011). Activated MMs play an important role in the pathological process of ischemic brain injury. Interestingly, MMs display extreme plasticity and can exhibit various activated phenotypes according to different microenvironments, to perform their multiple functions. Activated MMs mainly can be defined as two phenotypes, namely M1 pro-inflammatory phenotype and M2 anti-inflammatory phenotype (Hu et al., 2015; Du et al., 2017). It is known that M1-like MMs disrupt the blood-brain barrier (BBB) and worsen the neurological deficits by releasing inflammatory cytokines, such as tumor necrosis factor (TNF), interleukin 1 beta (IL-1β), and inducible nitric oxide synthase (iNOS) (Chen A. et al., 2019; Chen C. et al., 2019). In contrast, M2-like MMs clear cell debris, promote neurogenesis, angiogenesis, and axon regeneration, and release anti-inflammatory cytokines, such as arginase-1 (Arg-1), IL-10 and neurotrophic factors, thereby helping tissue repair (Hu et al., 2012; Nayak et al., 2014; Xiong et al., 2016; Lan et al., 2017). Besides, a recent study using a transcriptome analysis of microglia activation in a rat model of focal cerebral ischemia revealed that a vast majority of microglia are activated toward a wide spectrum of novel polarization states beyond the standard M1/M2 dichotomy, and especially associated with toll-like receptor (TLR) 2 and dietary fatty acid signaling pathways (Deng W. et al., 2020). Although the M1/M2 dichotomy has long served as the most common paradigm for microglia activation, it is worth noting that the M1/M2 phenotypes are an experimentally induced construct developed for *in vitro* study and are not representative of a binary response *in vivo*, but are often applied or described as a natural phenomenon (Ransohoff, 2016). While the M1/M2 dichotomy is an arbitrary construct, it can be a useful paradigm to simplify the discussion around MM polarization.

Recent studies have demonstrated that dynamic changes in the MM phenotype may be mediated by epigenetic modifications (Cheray and Joseph, 2018). Epigenetics refers to developmentally and environmentally induced heritable modifications that only affect gene expression without altering the DNA sequence. Epigenetic modifications mainly include histone post-translational modifications (such as methylation and acetylation), non-coding RNA (ncRNA) regulation, DNA methylation, nucleosome remodeling, and chromatin conformation changes (Allis and Jenuwein, 2016). Recently, there have been increasing evidence suggesting close associations of epigenetics with the pathological processes of cerebral ischemia (Kong et al., 2018). In addition, numerous studies have shed light on the relationship between epigenetic modifications and MM polarization. Histone modifications, ncRNA regulation, and DNA methylation have been identified to be involved in the transcriptional regulation of genes associated with microglial activation (Guo et al., 2016; Patnala et al., 2017; Rigillo et al., 2018; Wen et al., 2018; Li T. et al., 2020). However, the role of epigenetic modifications in MM polarization is understudied and its importance in ischemic stroke has just started to be elucidated.

Here, we first introduce the mechanisms by which epigenetic modifications regulate MM polarization. Afterward, we summarize epigenetic regulations of the MM polarization that contributes to neuronal cell death and the infarct development following ischemic stroke. Furthermore, we discuss key issues that require further research in epigenetic therapies focusing on MM polarization after ischemic stroke. This review presents the significant roles of MM polarization regulated by epigenetic modifications in cerebral ischemia and may contribute to the promotion of epigenetic modifications as an innovative diagnostic and therapeutic target for ischemic stroke.

## Epigenetic Regulations in Microglia/Macrophage Polarization

### Histone Modifications and Microglia/Macrophage Polarization

Histones are a group of conserved DNA-binding proteins that form the nucleosome, the basic unit of chromatin. Histones contain five components: H1, H2A, H2B, H3, and H4 (Ruthenburg et al., 2007). In addition to H1, the other four histones are combined as heterodimerization (total octamers), respectively, to form the core of the nucleosome. The core histones represent predominantly a globular C-terminal domain and a flexible N-terminal tail. These N-terminal tails of histones undergo post-translational modifications, including methylation, acetylation, phosphorylation, and ubiquitination (Kouzarides, 2007). These modifications of histone tails play crucial roles, such as affecting the structure and stability of chromatin by regulating the interaction of histones and DNA double strands and modulating the gene transcription through influencing the affinity of other transcription factors and structural gene promoters (Tessarz and Kouzarides, 2014). Currently, most of the information on histone modifications in MM polarization is methylation and acetylation at histone 3 and 4 (de Ruijter et al., 2003; Tang et al., 2014; Patnala et al., 2017; An et al., 2021).

#### Histone Acetylation and Microglia/Macrophage Polarization

Histone acetylation is dynamically regulated by histone acetyltransferases (HATs) and histone deacetylases (HDACs). HATs transfer the acetyl groups from the donor acetyl coenzyme A to the histone lysine residue. Reducing the charge on histones by these acetyl groups relaxes the interaction with DNA, resulting in a more open, accessible state, and thereby promoting gene transcription. HATs can be divided into type A and type B according to different sources and functions. Type A HATs, located in the nucleus, mainly acetylate nucleosome histones, are directly related to gene transcription regulation. While type B HATs, originally defined as cytoplasmic enzymes that acetylate free histones, have been proposed to play an important part in the assembly of chromatin through the acetylation of newly synthesized histones. In contrast, HDACs remove the acetylation modification of histone lysine residues, condense chromatin, and inhibit gene transcription (Wang et al., 2015).

Histone deacetylases have been proven to be crucial to the development and function of microglia. In the early prenatal period of microglia, the absence of hdac1 and hdac2, two class I HDACs, decreased the level of acetylation in the promoter region of pro-apoptotic and cell cycle genes, leading to increased apoptosis and decreased surviving microglia (Datta et al., 2018). In the AD mouse model, the depletion of HDAC1 and HDAC2 in MMs improved learning and memory by enhancing the amyloid phagocytosis of MMs and reducing the deposition of amyloid plaques (Li et al., 2019). In recent years, accumulating evidence suggests that HDAC inhibitors, including valproic acid, butyric acid, and trichostatin A, exert neuroprotective effects in many neurological diseases, such as cerebral ischemia (Kim et al., 2007; Li et al., 2019) and spinal cord injury (Zhang S. et al., 2018). It is also demonstrated that HDAC inhibitors can inhibit the activation of MMs. For instance, in microglia after lipopolysaccharide (LPS) treatment, the expression of HDACs increased. However, the treatment with either trichostatin A or suberoylanilide hydroxamic acid, increased H3 acetylation, attenuated the expression and the release of proinflammatory cytokines, TNF, IL-6, and iNOS, and suppressed the migration of microglia (Kannan et al., 2013). Additionally, valproic acid or butyric acid administration inhibited the activation of microglia and reduced the number of MMs in rats after middle cerebral artery occlusion (MCAO) (Kim et al., 2007). More importantly, a variety of HDAC inhibitors have been found to suppress the expression of pro-inflammatory cytokines induced by M1-like MMs, promote the expression of anti-inflammatory cytokines induced by M2-like MMs, and ultimately exert anti-inflammatory and neuroprotective effects (Hsing et al., 2015; Durham et al., 2017; Patnala et al., 2017; Chen et al., 2018; Li et al., 2019). The question to ask is how these HDAC inhibitors regulate the switch of the expression of pro-inflammatory and anti-inflammatory genes. This conversion may be related to the modulation of H3 deacetylation on the promoter of target genes. For example, in BV2 cells after being treated with LPS, the interaction between HDAC1 and the promoter of *G protein signaling (RGS) 10* was enhanced, which, in turn, inhibited H3 acetylation at the *RGS10* promoter, and consequently caused the decrease in the expression of *RGS10*, and thus elevated proinflammatory cytokine expression (Alqinyah et al., 2017). Moreover, the application of HDAC inhibitors increased the acetylation level of lysine 9 of histone 3 (H3K9) on *IL-10* gene in microglia, and accordingly promoted the transcription of *IL-10* and contributed to the transformation of microglia to M2-like phenotype (Patnala et al., 2017). Additionally, the inactivation of HDAC1 inhibited histones deacetylation on the promoter of *Krüppel-like factor 4 (KLF4)*, a zinc-finger transcription protein, and induced microglial polarization from M1-like to M2-like phenotype (Ji et al., 2019). At present, there are no reports on HATs regulating MM polarization, hence future studies need to address the exact role of HATs in regulating MM polarization.

#### Histone Methylation and Microglia/Macrophage Polarization

In addition to histone acetylation modifications, histone methylation also exerts a significant effect on modulating MMs phenotype. Histone methylation modifications occur mainly on lysine and arginine residues of histone (Shi and Whetstine, 2007). According to the number of modified methylation groups on histones, histone methylation can be divided into monomethylation, bimethylation, and trimethylation. Histone methylation is related to heterochromatin formation, gene imprinting, X chromosome inactivation, and the transcriptional regulation of genes (Martin and Zhang, 2005). Histone methylation is mainly catalyzed by histone methyltransferases (HMTs), which add methyl groups to histone lysine or arginine residues (Zhang, 2001; Husmann and Gozani, 2019). HMTs mainly include histone lysine methyltransferase, such as enhancer of zestehomolog 2 (EZH2) and histone arginine methyltransferase. In addition to HMTs, histone demethylases also synergistically regulate the balance of histone methylation by removing methyl groups at the amino terminus of histones (Klose and Zhang, 2007; Black et al., 2012). Histone demethylases mainly include lysine demethylase and jumonji domain (JMJD)-containing families (Klose and Zhang, 2007; Black et al., 2012). More importantly, methylation modifications at different sites of the histone may affect the transcriptional activity of the target genes. It is believed that methylation of lysine 4 (K4) of histone 3 (H3K4) and H3K36 often mediate the transcriptional activation of genes (Pfau et al., 2008), while methylation of H3K9 and H3K27 are associated with transcriptional repression (Soppe, 2002). Trimethylation on histone 3 lysine 27 (H3K27me3) is one of the most widely studied in MMs activation. The levels of H3K27me3 depend on the balance between two sets of histone-modifying enzymes, histone methyltransferase EZH2 and histone demethylase JMJD3 (Cheray and Joseph, 2018).

Enhancer of zestehomolog 2 is a component of polycomb repressive complex 2 (PRC2) and contains a SET (suvar, enhancer of zeste, trithorax) domain thought to be characteristic of methyltransferases which provide an active site for methylation reactions (Chase and Cross, 2011). Since EZH2 has no inherent enzymatic function, it needs to interact with other proteins in the PRC2, such as suppressor of zeste 12 and embryonic ectoderm development, to active its catalytic activity (Chase and Cross, 2011; Yang and Yu, 2013). Activated EZH2 catalyzes H3K27me3, resulting in target gene silencing (Chase and Cross, 2011; Melnick, 2012). Stimuli such as inflammation, injury, ischemia, and hypoxia increase the expression of EZH2. For example, LPS can rapidly increase the level of EZH2 mRNA in mouse primary microglial cells and BV2 cells (Arifuzzaman et al., 2017). In rats with neuropathic pain, the level of H3K27me3 increased, and the expression of EZH2 was upregulated in neurons and MMs in the spinal dorsal horn (Yadav and Weng, 2017). Strikingly, the expression of EZH2 increased in microglia after focal cerebral ischemia-reperfusion injury or oxygen glucose deprivation (OGD) (Chen J. et al., 2019). Elevated EZH2 induced by these stimuli was accompanied by activation of inflammatory genes, such as cytokines and chemokines. Accordingly, the administration of EZH2 inhibitors reduced the expression of cytokines and chemokines (Yadav and Weng, 2017), which, in turn, contributed to microglia polarizing toward the M2-like phenotype (Arifuzzaman et al., 2017; Chen J. et al., 2019). Similarly, it was confirmed that EZH2 deficiency in MMs resulted in the inhibition of the polarization of microglia to the M1-like phenotype in autoimmune encephalomyelitis mice (Zhang X. et al., 2018). In contrast to these reports, inhibition of EZH2 in glioblastoma has been found to upregulate iNOS and switch microglial polarization toward the M1-like phenotype, thereby enhancing phagocytosis of microglia and suppressing glioblastoma multiform tumor progression (Yin et al., 2017). However, the mechanism of the regulation of iNOS by EZH2 is still unknown. It is unclear whether EZH2 directly mediates the H3K27me3 at the iNOS promoter. These data highlight the significant role of histone methyltransferase EZH2 in regulating the polarization of MMs.

Enhancer of zestehomolog 2 can regulate microglial phenotype changes by the signal transduction and transcription activation factor 3 (STAT3) pathway. STAT3, a member of the STAT protein family of transcription factors, is a central signaling molecule that controls cellular response to environmental stimuli (Liang et al., 2016). The levels of phosphorylated STAT3 increased both in microglia of mice after MCAO and primary microglial cells after OGD (Chen J. et al., 2019). Phosphorylated STAT3 forms homo- or heterodimers, which bind to the specific sequences on the promoters of target genes and promote the transcription and expression of multiple genes encoding pro-inflammatory mediators, including cytokines, chemokines, adhesion molecules, and inflammatory enzymes (Yi et al., 2007). The pretreatment with 3-Deazaneplanocin A (DZNep), an EZH2 inhibitor, blocked the phosphorylation of STAT3, thereby decreasing the expression of genes related to the activation of M1-like microglia (Chen J. et al., 2019). However, there is little known about how EZH2 regulates the phosphorylation of STAT3. EZH2 may directly interact with STAT3 to phosphorylate STAT3 (Chen J. et al., 2019). Additionally, EZH2 can activate STAT3 by inhibiting suppressor of cytokine signaling 3 (SOCS3). SOCS3, a well-known anti-inflammatory mediator, has been shown to limit the excessive release of cytokines caused by persistent activation of STAT3 (Yoshimura et al., 2007). EZH2 specifically targets the promoter region of the SOCS3 gene and mediates the H3K27me3 at SOCS3 promoter, thereby suppressing the expression of SOCS3 (Zhang X. et al., 2018). Strikingly, in primary microglia with SOCS3 deficiency, the expression of activated STAT3 and pro-inflammatory cytokines increased, leading to microglia polarizing to the M1-like phenotype (Qin et al., 2012). The above studies reveal that EZH2 mediates the H3K27me3 of the promoter region of inflammatory mediators, such as SOCS3, thereby increasing the expression of inflammatory mediators and leading to MM polarization.

On the contrary, the histone demethylase JMJD3 promotes the polarization of M2-like microglia. A report showed that the expression of JMJD3 in healthy adult rat brain was enriched in microglia compared to neurons and astrocytes, while the level of global H3K27 methylation appeared to be lowest in microglia (Smith et al., 2014). After LPS treatment, the expression of JMJD3 mRNA or protein increased in microglia in both *in vivo* and *in vitro* studies (Alexaki et al., 2018). Moreover, the upregulation of JMJD3 blocked the expression of inflammatory genes associated with M1-like microglia, thereby promoting M2-like microglial polarization (Alexaki et al., 2018; Tao et al., 2019). In contrast, the inhibition of JMJD3 in N9 microglia suppressed M2-like microglial polarization and enhanced the inflammatory response (Tang et al., 2014). Interestingly, in cultured microglia, the administration of dehydroepiandrosterone elevated JMJD3, downregulated the H3K27me3 level, and promoted anti-inflammatory microglial activation after hemoglobin stimulation (Tao et al., 2019). These observations suggest a crucial role of JMJD3 in the polarization of microglia to the M2-like phenotype.

Little is known concerning the histone lysine demethylases enzymes in MMs. One study focused on JMJD2 [also known as lysine specific demethylase (KDM) 4A] which showed an upregulation in BV2 cells after the TLR3 and TLR4 stimulation (Das et al., 2015a). Moreover, after LPS treatment, KDM4A and KDM1A increased in microglia (Das et al., 2015b, 2016). Future studies should address the issues of how the histone lysine demethylases enzymes regulates MM polarization.

Although many studies have confirmed the crucial role of histone acetylation and methylation in regulating the polarization of MMs, there is no genome-wide analysis of MMs to address changes in histone methylation or acetylation levels. In contrast to the extensive investigations made on the effect of histone methylation and acetylation on MM polarization, few studies focused on the regulation of MM polarization by histone phosphorylation or ubiquitination.

### Non-coding RNAs and Microglia/Macrophage Polarization

NcRNAs are a type of RNA that do not encode proteins, but regulate gene expression at the post-transcriptional level. These ncRNAs can be divided into housekeeping ncRNAs and regulatory ncRNAs. The regulatory ncRNAs are classified as long non-coding RNAs (lncRNA) and small ncRNAs, which include microRNA (miRNA), short interfering RNAs, Piwi-interacting RNAs, and small nucleolar RNAs. There is a growing body of evidence that ncRNAs play a significant role in epigenetics. In this review, we will focus on miRNA and lncRNA and their epigenetic regulatory roles in MM polarization.

#### MicroRNAs and Microglia/Macrophage Polarization

MicroRNAs are a class of small ncRNAs composed of 22 nucleotides in length that mainly regulate gene expression at post-transcriptional levels (Ambros, 2004; Bartel, 2004). They can target 3′-untranslated region (3′-UTR) of mRNA and lead to the silence of target genes (Bi et al., 2009; Macias et al., 2009). It is reported that a total of 29 miRNAs were severely altered in the brain after hypoxia/ischemia and 6 of these miRNAs were involved in MM activation (Chen Y. et al., 2020). MiRNAs can modulate the polarization of MMs by directly regulating the transcription level of genes associated with MM activation. The effects of miRNAs on MM polarization are summarized in Table 1.

**TABLE 1:** The effects of miRNAs on microglia/macrophage polarization.

miRNA	Target	Phenotype	Inflammatory factors	Ref.
miR-325-3p	EGFR	M1 ↓	TNF, IL-1β↓	Chen Y. et al., 2020
miR-27a	TLR4	M1 ↓	IL-6, IL-1β, TNF, NO ↓	Yan et al., 2019
miR-374a-5p	SMAD6	M1 ↓	TNF, IL-1β↓	Lv et al., 2017
miR-200b	KLF4	M1 ↑, M2 ↓	iNOS, CD86, TNF, IL-1β↑; IL-4, IL-10, Arg-1, CD206 ↓	Chen Z. et al., 2020
miR-124	C/EBP-α	M1 ↓, M2 ↑	IL-1β, TNF ↓; IL-10, Arg-1 ↑	Ponomarev et al., 2011
	Arg-1	M2 ↑	Arg-1 ↑	Hamzei Taj et al., 2016a; Yu et al., 2017
	TLR4	M1 ↓, M2 ↑	IL-1β, IL-6, TNF, CD16 ↓; Arg-1, IL-4, IL-10, TGF-β, CD206 ↑	Hamzei Taj et al., 2016b
miR-575	PTEN	M2 ↑	Arg-1, CD206, Ym-1 ↑	Yang Y. et al., 2019
miR-26b	PTEN	M1 ↓, M2 ↑	IL-1β, TGF, CD32 ↓; Arg-1, CD206, Ym-1 ↑	Cheng et al., 2020

*EGFR: epidermal growth factor receptor; TNF: tumor necrosis factor; IL-1β, interleukin 1 beta; TLR4: toll-like receptor 4; IL-6: interleukin-6; NO: nitric oxide; SMAD6: mothers against decapentaplegic homolog 6; KLF4: Kruppel like factor 4; iNOS: induced nitrogen monoxide synthase; IL-4: interleukin-4; IL-10: interleukin-10; Arg-1: arginase-1; C/EBP-α: CCAAT/enhancer binding protein alpha; TGF-β: transforming growth factor-β; PTEN: phosphate and tension homology deleted on chromosome 10; ↑: positive regulation; ↓: negative regulation.*

MiR-325-3p, miR-27a, and miR-374a-5p have been described to inhibit the activation of MMs toward the M1-like phenotype and consequently reduced neuroinflammation. Yan et al. (2019) found that overexpression of miR-325-3p in LPS-induced BV2 cells negatively regulated the transcription of epidermal growth factor receptor (EGFR) by targeting its 3′-UTR, which in turn, inhibited the activation of microglia and the release of TNF and IL-1β. In addition, the expression of miR-27a in microglia declined rapidly after LPS stimulation. However, the transfection with miR-27a mimic in microglia inhibited the expression of TLR4 and interleukin-1 receptor-related kinase 4 (IRAK4) by directly binding the 3′-UTR of their mRNA, and therefore led to the inhibition of M1-like microglial polarization (Lv et al., 2017). Another study revealed that the transfection with miR-374a-5p agomir in primary microglia treated with LPS suppressed the activation of NOD-like receptor pyrin domain containing three (NLRP3) inflammasome signals by targeting smad6, a signal transducer and transcriptional modulator, and inhibited subsequent release of pro-inflammatory factors in M1 microglia (Chen Z. et al., 2020). In contrast to the extensive investigations on the miRNAs associated with the inhibition of M1-like MM polarization, few miRNAs have been proposed to be involved in the promotion of M1-like MMs. The study from Wen et al. (2018) showed that the overexpression of miR-200b induced microglial M1-like polarization in a rat model of MCAO by directly targeting KLF4 which binds to the promoter region of *Arg-1*, and accordingly reduced Arg-1 transcription. Future studies should address other miRNAs in promoting M1-like MM polarization.

A body of evidence confirmed that some miRNAs can promote the M2-like polarization of MMs. It has been found that miR-124 is the most abundant miRNA in neurons (Mishima et al., 2007). Interestingly, the resting phenotype of microglia is regulated by miR-124. In the steady state of the CNS, miR-124 may target the C/EBP-α/PU.1 pathway by binding to CCAAT/enhancer binding protein alpha (C/EBP-α) mRNA, and therefore inhibited the expression of C/EBP-α and maintained the resting phenotype of microglia (Ponomarev et al., 2011). Notably, recent studies have found that miR-124 promotes microglial M2-like polarization by downregulating C/EBP-α, thereby reducing the inflammatory damage caused by cerebral hemorrhage (Yu et al., 2017). There is evidence suggesting that intracerebral administration of miR-124 after ischemic stroke has been found to shift MMs into the anti-inflammatory phenotype by upregulating the expression of Arg-1 (Hamzei Taj et al., 2016a,b). Consistent with these findings, the exogenous miR-124 treatment promoted microglia toward M2-like polarization, hippocampal neurogenesis, and functional recovery by inhibiting the TLR4 pathway in rats after traumatic brain injury (TBI) (Yang Y. et al., 2019). In addition to miR-124, microRNA-575 was also reported to promote the M2-like polarization of MMs. For example, the increased expression of microRNA-575 induced by L-lysine downregulated phosphate and tension homology deleted on chromosome 10 (PTEN), thereby promoting the polarization of M2-like MMs and inhibiting the inflammatory response in mice with cerebral hemorrhage injury (Cheng et al., 2020). Interestingly, another miRNA, miRNA-26b was found to modulate the polarization of microglia through downregulating PTEN. The upregulation of miRNA-26b induced by glycine treatment resulted in the downregulation of PTEN, which in turn, activated Akt and inhibited SAH-induced M1-like microglial polarization (Qin et al., 2019). The relationship between miRNA-26b and M2-like MMs, however, needs further investigation.

#### Long Non-coding RNAs and Microglia/Macrophage Polarization

Like miRNAs, lncRNAs, a type of ncRNA with a sequence of more than 200 nucleotides, are also essential for the regulation of MMs polarization. LncRNAs can regulate gene expression at multiple levels, including the epigenetic, transcriptional, and post-transcriptional levels (Mercer and Mattick, 2013; Bär et al., 2016). LncRNAs can directly affect the polarization of MMs by regulating the expression of factors related to the polarization of MMs. Additionally, lncRNAs combine with miRNAs and indirectly regulate the transcription of genes, thereby affecting the phenotype of MMs.

LncRNA directly regulates the expression of transcription factors related to MM activation. The nuclear factor-kappa B (NF-κB) pathway is closely related to M1-like MM polarization. The inhibition of the NF-κB pathway has been considered to efficiently provide neuroprotection *via* suppressed M1-like MM activation (Popiolek-Barczyk et al., 2015; Ślusarczyk et al., 2018). Recent evidence has demonstrated that several lncRNAs have been involved in NF-κB pathway-mediated MM polarization. In a model of spinal cord injury and an LPS-treated BV2 cells, the expression of lncRNA metastasis-associated lung adenocarcinoma transcript 1 (MALAT1) was significantly increased, which was accompanied by an activation of IKKβ/NF-κB signaling pathway and an increase in the levels of TNF and IL-1β. Furthermore, the knockdown of MALAT1 attenuated M1-like microglia activation (Zhou et al., 2018). There are also reports showing the altered lncRNAs levels are closely related to NF-κB activation after cerebral ischemia or hypoxia. For instance, lncRNA Gm4419 expression has been found to increase during OGD injury of primary microglial cells. Moreover, Gm4419 is essential for promoting the phosphorylation of IkBα by binding to IkBα, resulting in increased translocation of NF-κB to the nucleus, thereby activating TNF, IL-1β, and IL-6, and contributing to OGD-induced inflammation by M1-like microglia (Wen et al., 2017). Zhang et al. (2019) illustrated that lncRNA 1810034E14Rik was significantly downregulated both in the cortex of MCAO mice and in the microglial cells induced by OGD or LPS. However, the overexpression of 1810034E14Rik inhibited the phosphorylation of p65 and subsequently increased mRNA levels of IL-10 and IL-4, but decreased mRNA levels of TNF and IL-1β, and alleviated inflammatory damage, suggesting that 1810034E14Rik can induce MM polarization from M1-like to M2-like phenotype by inhibiting NF-κB pathway (Zhang et al., 2019). In contrast to NF-κB, interferon regulatory factor (IRF) 4 as another important transcription factor is involved in the induction of the M2-like polarization of MMs (Lawrence and Natoli, 2011). It has been reported that lncRNA growth arrest-specific 5 (Gas5) was involved in regulating the expression of IRF4 and contributed to regulation of MM polarization. Mechanistically, Gas5 enhanced the interaction between EZH2 and *IRF4* promoter by combining with EZH2, which increased the methylation of H3K27 at the *IRF4* promoter and reduced the expression of IRF4, thereby inhibiting the M2-like MM polarization in experimental autoimmune encephalomyelitis C57BL/6 mice (Sun et al., 2017). In addition to the interaction with EZH2, lncRNAs regulate the polarization of MMs through other histone modifications, such as histone acetylation. Results from Wang et al. (2017a) showed that knockdown of lncRNA H19 downregulated HDAC1, thereby inhibiting the acetylation of histone H3 and H4, and blocking OGD-induced M1-like BV2 microglial polarization.

Long non-coding RNAs also function as a competing endogenous RNA and sponge miRNAs, thus regulating the expression of target mRNAs that regulate MM polarization. In other words, some lncRNAs hold miRNAs binding sites, which function as endogenous target mimics to bind with miRNAs and therefore block the interaction between miRNAs and their target genes (Wu et al., 2013). For example, both in mouse model of MCAO and BV2 cells treated with OGD, lncRNA small nucleolar RNA host gene 14 (SNHG14) attenuated the repression of miR-145-5p on the mRNAs of phospholipase A2 group IVA (PLA2G4A), which in turn, increased the expression of PLA2G4A, a pro-inflammatory pathway molecule, thereby promoting the microglia M1-like polarization (Qi et al., 2017). Another study showed that lncRNA tumor suppressor candidate 7 (TUSC7) could upregulate peroxisome proliferator-activated receptor gamma by decreasing the expression of miR449a, and lead to the suppression of microglial activation and inflammatory factors expression in HAPI cells after LPS stimulation (Yu et al., 2018).

Although many miRNAs and lncRNAs have been found to regulate MMs-mediated inflammation, only a few of them are involved in the polarization of MMs, which reflects the fact that little is known about the protein regulatory factors or transcription factors involved in this process. Moreover, since ncRNA is specific for different tissues and cell types, further studies are needed to gain an understanding of the potential role of other ncRNAs profiles in regulating MM polarization.

### DNA Methylation and Microglia/Macrophage Polarization

DNA methylation is well-known to mediate transcriptional silencing of downstream genes by recruiting suppressive transcription factors, which, in turn, leads to decreased gene expression. DNA methylation occurs on cytosine-phosphate-guanine (CpG) islands, the regions that are enriched with CG dinucleotides and are usually located at the promoter regions of genes, where cytosine at the 5′ end of CpG is converted into 5′-methyl-cytosine by DNA methyltransferases (DNMTs). In mammals, there are three main DNMTs: DNMT1, DNMT3a, and DNMT3b (Moore et al., 2013). DNMT1 is responsible for maintaining the methylation status of the genome, while DNMT3a and DNMT3b mediate the methylation of new CpG sites (Arand et al., 2012). In addition, DNA methyltransferases 3-like (DNMT3-like), a member of the DNMT3 family without DNA methyltransferase activity, interacts with DNMT3a and DNMT3b to activate their enzyme activities, thereby regulating *de novo* DNA methylation (Veland et al., 2019). Beside DNMTs, methyl-CpG-binding domain (MBD) proteins are also involved in the process of DNA methylation (Cronk et al., 2015). When CpG islands are methylated by DNMTs, MBD proteins are recruited to promoter 5′-methyl-cytosine to suppress gene transcription through interacting with numbers of partners, such as corepressor complexes (Jones et al., 1998; Esteller, 2007).

In contrast to the extensive investigations on the effects of histone modifications and ncRNA expression on MM polarization, the regulation of gene expression by DNA methylation is poorly studied in MMs. As mentioned above, miR-124 promotes MM polarization toward M2-like phenotype. Recent evidence suggests that the expression of miR-124 is regulated by DNA methylation, which impacts on MM polarization. For instance, the expression of DNA methylases such as DNMT1 and DNMT3a were upregulated in both rat primary microglia and BV2 cells treated with cocaine (Guo et al., 2016). These DNMTs targeted the promoter of primary miR-124 (pri-miR-124-1 and pri-miR-124-2) and increased the rate of CpG methylation in promoter region, which led to the downregulation of mature miR-124 (Guo et al., 2016). Comparable results were obtained with mouse primary microglia exposed to HIV-1 Tat (Periyasamy et al., 2018). In addition, methyl-CpG binding protein 2 (Mecp2) was found to suppress the expression of miR-124 in mouse primary microglial cells (Periyasamy et al., 2018). Mecp2, a member of the MBD family and a transcription repressor protein (Zhang et al., 2014), selectively binds to methylated DNA to silence specific gene promoters (Klose et al., 2005; Singh et al., 2008). Mecp2 should bind to methylated DNA in the promoter of miR-124, thereby inhibiting the expression of miR-124. On the other hand, HIV-1 tag-mediated downregulation of miR-124 increased the 3′-UTR target protein Mecp2, which blocked the miR-124 biogenesis, ultimately leading to further downregulation of miR-124 through a negative regulatory feedback axis and microglial activation (Periyasamy et al., 2018). Although these studies highlighted a critical role of DNMTs and MBD in regulating MM polarization, it is still unknown about which kind of DNMT plays the leading role. Hence, the exact isoform of DNMTs in the regulation of MM polarization needs further investigation.

## Epigenetic Dysregulation Is Involved in Microglia/Macrophage Polarization After Ischemic Stroke

When the body undergoes ischemic brain injury, insufficient blood supply leads to oxygen and glucose deprivation of neuronal cells in the ischemic area, and consequently causes cell damage. The injured cells then release neurotransmitters that have been previously produced and stored, such as free radicals, NO, and other toxic substances, which can quickly activate the MMs (Lee et al., 2014). Oxygen and glucose deprivation can also directly induce polarization of either BV2 cells or primary microglial cells (Wen et al., 2017; Ji et al., 2019; Zhang et al., 2019). It was observed in a study that the MMs around the infarct area underwent a dynamic polarization process in the MCAO model, as evidenced by the increase of the CD16/32 positive M1-like MMs around the infarct area at 3-14 days after MCAO. However, the number of CD206-positive M2-like MMs began to increase in the infarct area on Day 1 after MCAO, but gradually decreased after 1 week (Hu et al., 2012). This study reveals that the phenotype of MMs in the ischemic penumbra is different from that in the ischemic core area, suggesting that both the time and lesion of injury can affect the polarization of MMs after cerebral ischemia. Multiple factors and signal pathways have been identified to regulate MM polarization during cerebral ischemia. This section will describe epigenetic dysregulation of the MM polarization in ischemic stroke. A deeper understanding of epigenetics of MM polarization will help determine the epigenetic biomarkers and provide a promising treatment strategy for ischemic stroke.

### Roles of Microglia/Macrophage Polarization in Ischemic Stroke

Microglia/macrophage polarization could either exacerbate neuronal damage or promote neural repair after ischemic stroke. It is believed that M1-like MMs can prevent neurogenesis, inhibit angiogenesis, and destroy the integrity of white matter, while M2-like MMs can promote neurogenesis and angiogenesis, and maintain white matter integrity (Hu et al., 2015; Xiong et al., 2016; Lyu et al., 2021). There is plenty of evidence that M1-like microglia secrete pro-inflammatory cytokines, which can affect the proliferation and differentiation of neural stem/progenitor cells and impair neurogenesis (Butovsky et al., 2006). The administration of minocycline that selectively inhibits M1-like microglia (Kobayashi et al., 2013) can promote neurogenesis and functional recovery after focal cerebral ischemia (Liu et al., 2007). In addition, M1-like microglia can express pro-inflammatory factors such as iNOS and TNF, which may induce oligodendrocytes and white matter damage after cerebral ischemia (Deng et al., 2008; Uchida et al., 2010). Especially, an *in vitro* study showed that the treatment with conditioned medium from M1-like microglia aggravated oligodendrocytes death induced by OGD and inhibited the regeneration of oligodendrocytes (Wang et al., 2013). However, the promotion of MMs polarization to the M2-like phenotype protected oligodendrocytes from cerebral ischemia and maintained white matter integrity (Butovsky et al., 2006; Han et al., 2015; Qin et al., 2017). There are reports that perivascular M1-like microglia increase the release of TNF and induce endothelial necroptosis, thereby leading to destruction of the BBB (Jolivel et al., 2015; Chen A. et al., 2019). On the opposite side, targeting and regulating the polarization of MMs to M2-like phenotype can reduce cell apoptosis and enhance neurogenesis, which may become an important therapeutic strategy for cerebral ischemia (Xiao et al., 2020; Li et al., 2021). For example, M2-like MMs can also promote angiogenesis after focal cerebral ischemia, reduce infarct volume, and exert neuroprotective effects (Zhu J. et al., 2019; Shang et al., 2020). Moreover, the anti-inflammatory M2-like MMs promoted long-term neurovascular remodeling, thereby improving the neurological outcome after cerebral ischemia (Yang et al., 2015). Notably, the upregulation of matrix metalloproteinases after cerebral ischemia led to increased permeability of BBB, which recruited macrophages into the brain and thereby promoted tissue repair (Jiang X. et al., 2018; Ronaldson and Davis, 2020). These findings suggest that MM polarization might exert different functions after ischemic stroke.

### Epigenetic Regulations in Ischemic Stroke

Evidence increasingly points to the likelihood that a spectrum of epigenetic regulations, including histone modification, ncRNA, and DNA methylation, plays an important role in the pathophysiology of ischemic stroke. By altering transcriptional regulation, epigenetic regulations can exert influence on many pathways involved in the complex course of ischemic stroke, such as cell death, neuroinflammation, cerebral blood flow, neuronal regeneration, and neural repair and plasticity (Hu et al., 2017; Jhelum et al., 2017; Li et al., 2018; Ng et al., 2018; Stamatovic et al., 2019; Zhang and Wang, 2019; Kadir et al., 2020). More specifically, mutations and maladaptations of the epigenetic system on the level of histone acetylation, methylation, and DNA methylation are implied in cerebral ischemia (Kim et al., 2007; Baltan et al., 2011; Chen et al., 2012; Dock et al., 2015; Chen J. et al., 2019; Deng et al., 2019; Tang and Zhuang, 2019). For example, a general reduction of histone H3 and H4 acetylation levels in and around the ischemic core of the animal models of stroke has been identified (Ren et al., 2004; Kim et al., 2007; Xuan et al., 2012). Moreover, the application of HDAC inhibitors to restore the acetylation levels of H3 and H4 can reduce the infarct volume and improve the neurological outcome by maintaining the integrity of the BBB and anti-inflammatory effects (Ren et al., 2004; Langley et al., 2009; Liu et al., 2012; Li et al., 2019). The reduction levels of DNMT1 in post-mitotic neurons of mice has been demonstrated to alleviate ischemic brain injury (Endres et al., 2001). Furthermore, the pharmacological inhibition of DNA methylation ameliorates neurologic outcome in a rodent model of ischemia (Endres et al., 2001; Dock et al., 2015). A growing body of evidence reveals the miRNAs and lncRNAs profiles in the samples of blood (Li et al., 2015; Dykstra-Aiello et al., 2016; Zhu W. et al., 2019; Bejleri et al., 2021) and cerebrospinal fluid (Sørensen et al., 2014) from patients with ischemic stroke. These ncRNAs are involved in multiple pathological processes of stroke and could be used as biomarkers for the diagnosis and prognosis of ischemic stroke (Tiedt et al., 2017; Li et al., 2018; Heydari et al., 2020; Stanzione et al., 2020; Bejleri et al., 2021). Notably, preliminary data indicate that neuroprotective agents targeting epigenetic regulations can modulate neural cell regeneration and promote brain repair and functional recovery after cerebral ischemia (Dock et al., 2015; Mitić et al., 2015; Wang et al., 2015; Alexaki et al., 2018; Brookes et al., 2018; Chen J. et al., 2019; Jaworska et al., 2019; Ji et al., 2019). Therefore, a better understanding of how epigenetic regulations influence the process of cerebral ischemia will pave the way for discovering new targets and therapeutics for ischemic stroke.

### Histone Acetylation and Microglia/Macrophage Polarization in Ischemic Stroke

Recently, the role of histone acetylation in ischemic stroke has attracted much attention. Dysregulated histone acetylation was observed in animal models of ischemic stroke. Studies both in transient global and focal cerebral ischemia model of rats revealed a decreased expression of acetylation of H3 and H4, which was related to severe brain injury (Ren et al., 2004; Xuan et al., 2012). Especially, in the mouse MCAO model, the levels of acetylation of H3K9 were downregulated in activated MMs cells in the cerebral cortex, striatum, and hippocampus (Patnala et al., 2017). Surprisingly, growing evidence indicates that the administration of HDAC inhibitors was found to reduce brain damage and improve the prognosis of stroke through remission of inflammation, thus promoting neurogenesis and functional restoration. For example, the treatment with butyric acid blocked the upregulating acetylation of H3K9 in the *il10* promoter, which contributed to the increase in *il10* gene transcription and protein levels. The upregulation of IL10 induced by butyric acid in MMs switched its phenotype from M1-like to M2-like and promoted neurogenesis and mitigated MMs-mediated neuroinflammation (Patnala et al., 2017). Similarly, the treatment with HDAC inhibitors, such as valproic acid, butyric acid, or trichostatin A, can prevent the decrease of acetylated H3 levels in the ischemic brain, reduce the volume of cerebral infarction, and improve the motor, sensory, and reflex ability of permanent MCAO rats (Kim et al., 2007). Additionally, there is also evidence supporting the anti-inflammatory effect of butyric acid in a transient MCAO mouse model (Li et al., 2019) and promotion of neurogenesis of butyric acid in a neonatal rat model of hypoxia-ischemia (Jaworska et al., 2019). Although significant progress has been made in understanding the pathogenesis and treatment of HDAC inhibitors in cerebral ischemia-reperfusion injury from preclinical studies, clinical studies of HDAC inhibitors in ischemic stroke patients are limited. A recent clinical study revealed that inhibiting HDAC9 might be a target for secondary prevention of ischemic stroke (Brookes et al., 2018). In Brookes et al. (2018)’s study, the authors recruited patients with previous ischemic stroke or transient ischemic attack and found that these patients treated with sodium valproate, a non-specific inhibitor of HDAC9, had a lower risk of recurrent stroke.

### Histone Methylation and Microglia/Macrophage Polarization in Ischemic Stroke

In contrast to the extensive studies conducted on the role of HDACs on MM activation after cerebral ischemia, the effect of histone methylation on MM activation after cerebral ischemia remains unknown. The expression of the histone methyltransferase EZH2 was upregulated both in the peri-infarct area of mice after MCAO and in microglia after OGD (Gao et al., 2006; Chen J. et al., 2019). The administration of DZNep, the EZH2 inhibitor, prevented the activation of M1-like MMs, reduced the infarct volume, and improved behavioral performance. Additionally, a recent study indicated that the treatment with DZNep regulated the expression of pro-angiogenic genes in endothelial cells, and increased angiogenesis in mouse ischemic limb muscles, which might contribute to the repair of ischemic limbs (Mitić et al., 2015). These studies indicate that inhibiting EZH2 may restrain the polarization of M1-like MMs and promote angiogenesis. However, few studies focused on the changes of histone methylation after ischemic stroke. Gao et al. (2006) demonstrated that the methylation level of H3 decreased in the hippocampal CA3 region in the neonatal rat after brain injury. Nevertheless, the relationship between histone methylation and MM polarization in ischemic stroke still needs further exploration.

### MicroRNAs and Microglia/Macrophage Polarization in Ischemic Stroke

It is noteworthy that miRNAs have a high abundance in the CNS, and mostly their expression patterns are brain specific. MiRNAs participate in the pathophysiological process of ischemic stroke, including excitotoxicity, oxidative stress, cell apoptosis, glia activation, and neuroinflammation (Li et al., 2018). It appears, therefore, miRNAs can be potential diagnostic and prognostic biomarkers for ischemic stroke, while some miRNAs could be further developed into therapeutic targets.

MicroRNAs serve as biomarkers for the diagnosis of ischemic stroke. Sonoda et al. (2019) conducted a stroke risk prediction for 1523 healthy people and identified seven differentially expressed miRNAs after ischemic stroke and then determined that the serum signal values of these miRNAs were related to the incidence or prevalence of ischemic stroke. There are also reports identifying a group of circulating miRNAs, such as miR-125a-5p, miR-125b-5p, and miR-143-3p, associated with acute ischemic stroke (AIS), which can be used as early diagnostic indicators (Tiedt et al., 2017). Another study demonstrated that the expression of let-7e was the lowest in the recovery phase but the highest in the acute phase of AIS, suggesting that the level of let-7e in the serum may serve as a circulating biomarker for the acute stage of AIS (Peng et al., 2015). Interestingly, 6 serum miRNAs, miR-125b, miR-125a, let-7b, let-7e, miR-7-2-3p, and miR-1908, evaluated by a systematic gene chip study, were reported to be differentially expressed in diverse ischemic stroke subtypes (Gui et al., 2019), suggesting that circulating miRNAs could be involved in the pathogenesis of ischemic stroke, and potentially be novel diagnostic biomarkers for ischemic stroke subtypes.

Several miRNAs are believed to have contributed to the improvement of motor functional outcome after stroke. The injection of lentiviral miR-126-3p or -5p decreased brain infarct volume and edema volume at 3 days after cerebral ischemia (Ni et al., 2015; Pan et al., 2020). Particularly, miRNAs may improve the prognosis of stroke by regulating the polarization of MMs. For instance, the mice treated with the let-7c-5p mimic exhibited smaller infarct volumes in ipsilateral cortex and striatum and had better performance in the corner and rotarod tests after MCAO by inhibiting the inflammation mediated by M1-like MMs (Ni et al., 2015). Another study demonstrated that miR-669c overexpression elevated Arg1 levels in the ischemic brain of mice and improved sensorimotor functions (Kolosowska et al., 2020).

Some miRNAs are potential therapeutic targets for ischemic stroke. In a mice model of cerebral ischemia, miR-155 was found to be upregulated in the injured cerebral cortex (Ma et al., 2020). The inhibition of miR-155 expression through intraperitoneal injection with resveratrol after cerebral ischemia resulted in M2-like polarization of MMs and reduced neuroinflammation (Ma et al., 2020). In addition, miR-424 and miR-210 were reported to be related with oxidative stress after stroke. The overexpression of miR-424 has been shown to reduce oxidative stress, thereby protecting the brain from ischemic damage (Zhao et al., 2013). The treatment with agomiR-424 increased the expression of the antioxidant factor nuclear factor erythroid 2 related factor and reduced the infarct volume (Shih, 2005; Liu et al., 2015). Similarly, lentiviral overexpression of miR-424 inhibited the activation of M1-like MMs and neuronal apoptosis and decreased cerebral infarction size and brain edema after MCAO in mice (Zhao et al., 2013). Another study demonstrated that the knockout of miR-210 can reduce the death of cortical neurons and the oxidative stress response of vagus nerve stimulation after transient MCAO (Jiang et al., 2015). Also, the administration of miR-210 inhibitors effectively reduced hypoxic-ischemic encephalopathy damage by inhibiting MM M1-like activation (Li B. et al., 2020). More importantly, silencing miR-210 expression significantly decreased cerebral infarction volume and brain edema, and ameliorated behavioral deficits, although the study failed to show the relationship between these neuroprotective effects of miR-210 silence with the suppression of M1-like MMs (Huang et al., 2018).

Notably, recent studies showed that exosomes carrying miRNAs regulate MM polarization after cerebral ischemia. Exosomes, tiny vesicles with a lipid bilayer membrane, can be secreted by most cells. Since the diameter of exosomes is only 30–150 nm, they can penetrate the BBB and be detected in peripheral blood or cerebrospinal fluid. Exosomes function as a key participant in mediating intercellular communication through transferring proteins and ncRNAs between cells (Kalluri and LeBleu, 2020). The transport of miRNAs mediated by exosomes has received increasing attention in the treatment of ischemic stroke. For example, the treatment with miR-30d-5p-rich exosome can suppress the inflammatory response and reverse ischemia-induced brain injury (Jiang M. et al., 2018). Similarly, recent research demonstrated that the administration of exosomes miR-26b-5p suppresses the M1-like MM polarization and reduces neuronal damage after brain ischemia/reperfusion (Li G. et al., 2020). Interestingly, miRNA array data showed that the exosomes derived from M2 microglia are rich in miR-124 (Song et al., 2019). More remarkably, the treatment of exosomes derived from M2-like microglia significantly increased the cell surviving and decreased neuronal apoptosis in primary cortical neurons after OGD, and significantly reduced cerebral infarct volume and improved behavioral disorders in mice 3 days after focal cerebral ischemia (Song et al., 2019). These studies indicate that exosomes carrying miRNAs may become new targets for the treatment of ischemic stroke by regulating MM polarization.

### Long Non-coding RNAs and Microglia/Macrophage Polarization in Ischemic Stroke

In addition to miRNAs, the roles of lncRNAs in ischemic stroke have been implicated. Dykstra-Aiello et al. (2016) collected whole blood RNA samples from 266 patients with ischemic stroke and evaluated the expression of lncRNAs in these samples. They found the differentially expressed lncRNAs between ischemic stroke patients and controls, which suggests that lncRNAs have enormous potential to be used as novel clinical biomarkers for stroke (Dykstra-Aiello et al., 2016). More interestingly, Wang et al. (2017a) found that lncRNA H19 gene mutations increase the risk of ischemic stroke.

Recently, studies have found that lncRNAs TUG1, SNHG14, H19, Gm4419, and 1810034E14Rik are associated with the prognosis of ischemic stroke by regulating cell apoptosis and inflammation. Emerging studies have illustrated that lncRNA taurine upregulated gene 1 (TUG1) is upregulated after ischemia-reperfusion injury in many organs, such as brain, heart, kidney, and spinal cord (Jia et al., 2019; Yang D. et al., 2019; Shan et al., 2020; Xu et al., 2020a). Overexpression of TUG1 promoted neuronal apoptosis in mice after MCAO (Xiong et al., 2018), and knockout or silencing of the *TUG1* gene reduced apoptosis and promoted cell survival (Xiong et al., 2016; Chen et al., 2017). Importantly, the expression of TUG1 was upregulated in BV2 microglia after OGD (Dock et al., 2015). Knockout of the *TUG1* gene promoted the transformation of microglia from M1-like to M2-like phenotype and accordingly promoted survival of SH-SY5Y cells (Wang et al., 2019). These studies suggest that TUG1 may affect cell apoptosis by regulating the polarization of microglia, thus improving the prognosis of ischemic stroke. There is evidence supporting an upregulation of SNHG14 in cerebral ischemia-reperfusion injury and hypoxia-reoxygenation-induced neurons or BV2 cells (Qi et al., 2017; Deng Z. et al., 2020). SNHG14 overexpression promoted apoptosis in HT22 cells induced by OGD (Chen et al., 2012). Similarly, SNHG14 may increase the apoptosis of neurons by promoting the M1-like polarization of BV2 microglia (Qi et al., 2017). In addition, shRNA-mediated silencing of SNHG14 alleviated neuronal impairment and inflammation in response to OGD in PC-12 cells (Zhong et al., 2019). Also, H19, another lncRNA, is closely related to cell apoptosis after ischemic stroke. In BV2 cells, the knockout of the *H19* gene promoted cell proliferation, reduced cell apoptosis, and ameliorated inflammation after OGD (Wang et al., 2017a,b; Wen et al., 2018). More profoundly, silencing or knockout of the *H19* gene can improve the neurological outcome in the rodent MCAO stroke model (Wang et al., 2017b). Further, the H19 gene knockout inhibited the activation of M1-like microglia but promoted the polarization of M2-like microglia in BV2 cells after OGD (Wang et al., 2017b). Therefore, the inhibition of H19 may improve the prognosis of ischemic stroke by promoting the polarization of microglia to M2-like and reducing neuronal apoptosis. LncRNAs can also modulate inflammation to affect the prognosis of ischemic stroke. It has been found that the upregulated Gm4419 in primary microglial cells after OGD induced the activation of M1-like microglia (Wen et al., 2017). Contrary to Gm4419, the overexpression of lncRNA-1810034E14Rik significantly decreased TNF and IL-1β, but increased IL-4 and IL-10 production and secretion in primary microglial cells after OGD (Zhang et al., 2019). Also, the overexpression of 1810034E14Rik played an anti-inflammatory role and improved the motor function of mice after MCAO (Zhang et al., 2019).

Taken together, circulating ncRNAs are differentially expressed after ischemic stroke. These differentially expressed ncRNAs might be used for biomarkers for the diagnosis and prognosis of ischemic stroke. In addition, ncRNAs can function as central regulators of pathological processes related with MM polarization during ischemic stroke, which makes ncRNAs potential targets for stroke treatment. However, more work needs to be done to better delineate the involvement of specific ncRNAs in ischemic stroke and to explore their utility for clinical applications.

### DNA Methylation and Microglia/Macrophage Polarization in Ischemic Stroke

Cerebral ischemia leads to dynamic changes in DNA methylation. The global level of DNA methylation is higher in animal models with ischemic stroke (Endres et al., 2001). However, the lower blood level of DNA methylation may be related to the higher ischemic stroke risk. Mechanistically, studies have found that the increased levels of VCAM-1, a protein that promotes blood vessel-immune cell interaction and mediates atherosclerosis, are associated with the hypomethylation of long interspersed nucleotide elements 1, leading to the high occurrence of cardiovascular and cerebrovascular diseases in the elderly (Ridker, 2001; Baccarelli et al., 2010). In addition, reduction of DNA methylation through pharmacological inhibition of DNMTs activity or genetic deletion of DNMTs is associated with better neurological outcome after ischemic stroke. For example, the genetic deletion of DNMTs or treatment with DNMTs inhibitor 5-aza-2′-deoxycytidine was shown to reduce cerebral infarct size in striatum and ischemic brain damage (Endres et al., 2001). Another study revealed that the reduction of DNMT1 decreased delayed neuronal death in the hippocampal CA1 region of gerbils after transient cerebral ischemia (Endres et al., 2000). However, how DNA methylation modulates MM polarization after ischemic stroke remains unknown. Few studies have emphasized the key role of DNMTs in regulating the polarization of MMs (Eskey et al., 2018; Periyasamy et al., 2018). One study reported that DNMT3L was increased in BV2 microglia treated with LPS (Das et al., 2015b). Nevertheless, there are no studies yet investigating the changes of DNMTs in MMs after cerebral ischemia or the roles of DNMTs in MM polarization following cerebral ischemia.

## Conclusion

In this review, we have emphasized the molecular mechanisms of epigenetic regulation, including histone modifications, ncRNA regulation, and DNA methylation, involved in regulating MM polarization. Furthermore, we proposed that epigenetic dysregulation in MM polarization contributes to neuronal death and the development of functional impairment after ischemic stroke. Components of the epigenetic machinery, such as DNMTs, HDACs, miRNAs, and lncRNAs, all represent potential targets for the development of epigenetic drugs. DNA methylation inhibitors and HDAC inhibitors may be the most promising drugs to regulate microglia activation after ischemic stroke. Some members of them have been approved by the United States Food and Drug Administration for the treatment of hematological malignancies and cancer. Multiple studies conducted in rodent models of stroke have shown the neuroprotective and neuroregenerative effects after the treatment of HDAC inhibitors. However, most drugs that target epigenetic mechanisms are non-specific. For example, HDAC inhibitors and DMNT inhibitors are not selective for brain regions and cell types (Xu et al., 2020b). Non-specific HDAC inhibitors may cause a variety of adverse reactions, including weight loss, dysgeusia, electrolyte changes, and arrhythmia (Kao and Lin, 2019). So, it is more appropriate to choose epigenetic drugs that target specific epigenetic modifications rather than affect global modifications. In addition, due to the physiological barrier effect of the BBB, it is necessary to develop epigenetic drugs that can penetrate the BBB. Considering that epigenetic changes in ischemic stroke may be the result of simultaneous regulation of multiple genes, and there may be interactions between epigenetic networks, in the future it is necessary to develop epigenetic editing tools with multiple genomic sites that can simultaneously target different epigenetic markers. NcRNA, such as miRNAs and lncRNAs, likely represent both new biomarkers and new therapeutic targets for ischemic stroke in the future. A variety of miRNAs and lncRNAs have been observed to undergo changes in peripheral blood samples of patients with acute stroke as well as chronic time points, and they might provide new avenues to serve as biomarkers for rapid diagnosis and treatment efficacies in ischemic stroke. However, the miRNAs and lncRNAs that play a central role in MM polarization have not yet been determined. The key miRNA or lncRNA participants in microglia activation may have similar roles in other pathological environments, so a larger amount of preclinical and clinical studies is still needed to determine specific epigenetic markers that regulate MM polarization after ischemic stroke. Moreover, single-cell sequencing technology and proteomic studies can provide insight into the differences in microglial responses to cerebral ischemia between and/or within regions of the brain to obtain specific epigenetic markers. In conclusion, epigenetic regulations serve as important regulators of MM polarization after ischemic stroke. Epigenetic dysregulation is involved in MM polarization after ischemic stroke and might be used for diagnosis and prognosis to guide clinical decision-making. However, more work is needed to better delineate the involvement of epigenetic regulations of MM polarization in stroke and to exploit their utility for clinical applications.

## Author Contributions

This work was primarily written by MQ and LZ. LZ contributed to the overall conceptual design and creation of this work. EX assisted with manuscript revision. All authors read and approved the final manuscript.

## Conflict of Interest

The authors declare that the research was conducted in the absence of any commercial or financial relationships that could be construed as a potential conflict of interest.

## Publisher’s Note

All claims expressed in this article are solely those of the authors and do not necessarily represent those of their affiliated organizations, or those of the publisher, the editors and the reviewers. Any product that may be evaluated in this article, or claim that may be made by its manufacturer, is not guaranteed or endorsed by the publisher.

## Abbreviations

MMs: microglia/macrophages
CNS: central nervous system
TNF: tumor necrosis factor
IL: interleukin
IL-1 β: interleukin 1 beta
iNOS: inducible nitric oxide synthase
Arg-1: arginase-1
ncRNA: non-coding RNA
HATs: histone acetyltransferases
HDACs: histone deacetylases
MCAO: middle cerebral artery occlusion
LPS: lipopolysaccharide
RGS: G protein signaling
H3K9: lysine 9 of histone 3
KLF4: Krüppel-like factor 4
HMTs: histone methyltransferases
EZH2: enhancer of zestehomolog 2
JMJD: jumonji domain
K4: lysine 4
H3K4: lysine 4 of histone 3
H3K36: lysine 36 of histone 3
H3K27: lysine 27 of histone 3
H3K27me3: trimethylation on histone 3 lysine 27
PRC2: polycomb repressive complex 2
SET, suvar: enhancer of zeste trithorax
OGD: oxygen glucose deprivation
STAT3: signal transduction and transcription activation factor 3
DZNep: 3-deazaneplanocin A
SOCS3: suppressor of cytokine signaling 3
KDM: lysine specific demethylase
TLR: toll-like receptor
miRNA: microRNA
lncRNA: long non-coding RNA
3′-UTR: 3′-untranslated region
EGFR: epidermal growth factor receptor
IRAK4: interleukin-1 receptor-related kinase 4
NLRP3: NOD-like receptor pyrin domain containing three
C/EBP- α: CCAAT/enhancer binding protein alpha
TBI: traumatic brain injury
PTEN: phosphate and tension homology deleted on chromosome 10
NF- κ B: nuclear factor-kappa B
MALAT1: metastasis-associated lung adenocarcinoma transcript 1
IRF: interferon regulatory factor
Gas5: growth arrest-specific 5
SNHG14: small nucleolar RNA host gene 14
PLA2G4A: phospholipase A2 group IVA
TUSC7: tumor suppressor candidate 7
CpG: cytosine-phosphate-guanine
DNMTs: DNA methyltransferases
DNMT3L: DNA methyltransferases 3-like
Mecp2: methyl-CpG binding protein 2
BBB: blood-brain barrier
TUG1: taurine upregulated gene 1.
